# Antioxidant enzymes, oxidative stress, and physiological aging markers in *Drosophila melanogaster* (Diptera: Drosophilidae): a systematic review with translational perspectives

**DOI:** 10.1093/jisesa/ieag020

**Published:** 2026-03-24

**Authors:** Anđela Savić, Mirjana Šipovac, Aleksandra Novaković, Ljubica Vasiljević, Maja Palangetić

**Affiliations:** Faculty of Technology Zvornik, University of East Sarajevo, Zvornik, Bosnia and Herzegovina; Faculty of Technology Zvornik, University of East Sarajevo, Zvornik, Bosnia and Herzegovina; Faculty of Education, University of East Sarajevo, Bijeljina, Bosnia and Herzegovina; Faculty of Technology Zvornik, University of East Sarajevo, Zvornik, Bosnia and Herzegovina; Faculty of Technology Zvornik, University of East Sarajevo, Zvornik, Bosnia and Herzegovina

**Keywords:** oxidative stress, antioxidant enzymes, lifespan, locomotor activity, translational research

## Abstract

Oxidative stress contributes to cellular damage and aging. In this study, the model organism *Drosophila melanogaster* Meigen 1830 was used to examine the molecular and physiological mechanisms of aging associated with oxidative stress. A systematic search and detailed analysis of scientific publications on this topic were conducted using the PubMed database. The analysis included 30 original research articles published between April 2020 and April 2025. Studies focusing primarily on upstream signaling pathways or other stress mechanisms without direct measurement of reactive oxygen species (ROS), antioxidant enzymes, or physiological aging markers were excluded. For each article, key elements were extracted, including the research topic, assessed ROS and antioxidant enzyme markers, physiological aging indicators, main experimental findings, and study conclusions. The results were classified using a descriptive frequency-based analysis of reported ROS and aging markers, as well as by the main topic and research approach of each study. Collectively, the reviewed studies indicate that excessive ROS production leads to oxidative stress and lipid damage, particularly in older individuals. The enzymes superoxide dismutase and catalase were the most commonly assessed markers and served as primary indicators of oxidative homeostasis. Lifespan and locomotor activity were identified as the main physiological aging parameters, while natural extracts and phytonutrients were the most frequently used intervention agents. These findings confirm the value of *D. melanogaster* as a model for aging research and support its use in identifying conserved redox mechanisms and evaluating potential interventions with translational relevance for human health.

## Introduction

Aging is a dynamic process characterized by the gradual and cumulative damage to cells, progressive decline in cellular function, and increased susceptibility to morbidity (Valavanidis et al. 2012). The aging process also involves molecular-level changes, including genomic instability, telomere attrition, epigenetic alterations, and proteostasis loss (López-Otín et al. 2013). Cellular senescence refers to the irreversible loss of proliferative capacity, typically occurring after multiple divisions in culture. Senescent cells undergo various alterations, including secretory changes, and are thought to accumulate in aging tissues, playing a key role in the initiation and progression of aging (Jeyapalan et al. 2007, Wang et al. 2009). The oxidative stress theory of aging was first proposed in 1956 by American scientist Denham Harman (2006). This theory was later refined with the discovery of superoxide dismutase (SOD), an enzyme involved in the *in vivo* conversion of superoxide radicals ($O_{2}^{•-}$) into hydrogen peroxide (H_2_O_2_) and molecular oxygen (O_2_), representing a major cellular antioxidant defense mechanism. Oxidative stress is widely recognized as a central contributor to cellular senescence and age-related functional decline, largely through elevated levels of reactive oxygen species (ROS). Senescent cells acquire an irreversible senescence-associated secretory phenotype characterized by the secretion of soluble factors (interleukins, chemokines, and growth factors), degradative enzymes, and insoluble extracellular matrix components (Akshaj et al. 2016, Chandrasekaran et al. 2017).

Oxidative stress, cellular senescence, and associated structural phenotypic changes play central roles in the development and progression of various acute and chronic pathological conditions. These include acute and chronic kidney disease, neurodegenerative disorders, ocular diseases, biliary conditions, and several types of cancer (Marquez-Exposito et al. 2022, Böhm et al. 2023, Jin et al. 2024, Castelli et al. 2025). In particular, cardiovascular risk factors such as obesity, diabetes, hypertension, and atherosclerosis are linked to chronic cytokine-mediated inflammation, further accelerating cellular senescence (Chandrasekaran et al. 2017). In neurodegenerative diseases, especially Alzheimer’s disease, increased levels of senescence markers such as p16, matrix metalloproteinases, and the inflammatory cytokine IL-6 have been detected in brain tissues, suggesting a strong connection between chronic inflammation, cellular senescence, and neurodegeneration (Burton et al. 2010).

There is a close interplay between oxidative stress, inflammation, and aging, as aging reflects a loss of homeostasis due to chronic oxidative stress, which particularly affects regulatory systems such as the nervous, endocrine, and immune systems. This results in activation of inflammatory pathways, perpetuating chronic inflammation and creating a vicious cycle in which oxidative stress and inflammation reinforce each other, ultimately contributing to increased age-related mortality (De la Fuente and Miquel 2009).

*Drosophila* species (Diptera: Drosophilidae) are widely used in gerontology research due to several experimental advantages: high reproductive capacity, short generation time, small body size, ease and low cost of cultivation, a small number of chromosomes, and the availability of numerous well-characterized and mapped genetic mutations. Among them, *Drosophila melanogaster* stands out as the most extensively studied and genetically tractable model, particularly suited for aging research due to its short lifespan and highly conserved biological pathways relevant to oxidative stress and cellular senescence (Piper and Partridge 2018). Investigating aging through the lens of oxidative stress using *D. melanogaster* thus provides valuable insight into the molecular basis of lifespan regulation and healthspan maintenance.

Despite a growing number of studies investigating oxidative stress in *D. melanogaster*, there is a notable lack of integrative analyses that combine biochemical markers (eg SOD, catalase (CAT), and malondialdehyde (MDA)) with functional indicators (eg locomotor activity, gut homeostasis, and stress resistance). Moreover, the influence of variables such as sex, dose, and the timing of intervention remains insufficiently characterized. Addressing these gaps through systematic synthesis is essential for designing effective antiaging strategies and understanding conserved mechanisms of redox regulation.

In the following sections, we first provide definitions and mechanisms of oxidative stress and ROS formation and then discuss antioxidant defenses, oxidative damage to macromolecules, and their impact on cellular and organismal aging in *Drosophila*.

### ROS and Oxidative Stress Mechanisms

Under normal physiological conditions, ROS are continuously produced as by-products of cellular respiration and other metabolic redox reactions. At physiological levels, ROS serve as signaling molecules in immune defense and redox regulation; however, excessive accumulation disturbs redox homeostasis and causes oxidative stress. This imbalance leads to mitochondrial dysfunction, DNA and protein oxidation, lipid peroxidation, and telomere shortening, which collectively contribute to cellular damage and aging (Moskovitz and Oien 2010, Moskalev et al. 2013, Ayala et al. 2014, Margaritis et al. 2017). ROS can exist as free radicals or as nonradical species capable of generating radical intermediates. Free radicals are by-products of normal cellular metabolism and are characterized by the presence of 1 or more unpaired electrons, making them unstable and extremely reactive (Kozlov et al. 2024). Due to this reactivity, they extract electrons from other molecules, initiating chain reactions that damage cellular components.

Free radicals are typically short-lived and highly reactive, often unable to diffuse far from their site of generation before being neutralized. Examples of radical ROS include $O_{2}^{•-}$, hydroxyl radical (^•^OH), peroxyl radical (ROO^•^), hydroperoxyl radical (${\text{HO}}_{2}^{•}$), and alkoxyl radicals (RO^•^). In contrast, nonradical ROS lack unpaired electrons, are generally less reactive, and may diffuse from their site of origin. Nonradical ROS include H_2_O_2_, organic hydroperoxides (ROOH), and other partially reduced oxygen species (Phaniendra et al. 2015). Despite their lower reactivity, nonradical ROS remain partially reduced species capable of participating in redox reactions that can subsequently generate free radicals (Travasso et al. 2017). Notably, H_2_O_2_ can freely diffuse across cellular compartments, making it particularly relevant in redox signaling and antioxidant distribution within different subcellular domains (Coling et al. 2009, Di Marzo et al. 2018). At moderate levels, ROS can be beneficial, playing roles in cellular signaling, immune defense, and redox regulation (Averill-Bates 2024, Kozlov et al. 2024).

### Antioxidant Defense System: Key Enzymes and Molecules

The term antioxidant refers to a broad class of molecules—including bioactive substances and enzymatic complexes—that, even in small amounts, can protect both natural (eg phospholipids, proteins, DNA) and artificial substrates (eg plastics, oils) from free radical-induced damage. Antioxidants that reduce or inhibit the formation of ROS are categorized as preventive substances, as they act to inhibit the formation of radical initiators (Tumilaar et al. 2024).

**Fig. 1.**
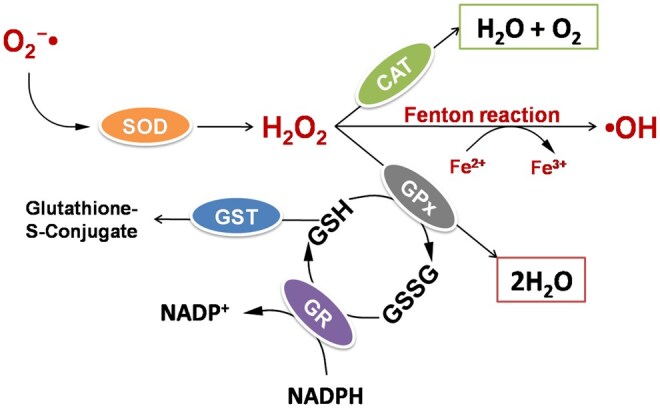
Schematic representation of antioxidant defense enzymes counteracting reactive oxygen species (ROS). The diagram highlights enzymatic antioxidant defense mechanisms involved in ROS detoxification. Superoxide dismutase (SOD) converts superoxide radicals ($O_{2}^{•-}$) into hydrogen peroxide (H_2_O_2_). Catalase (CAT) decomposes H_2_O_2_ into water and oxygen, whereas glutathione peroxidase (GPx) reduces H_2_O_2_ to water. Glutathione S-transferase (GST) contributes to detoxification through conjugation reactions. Reduced glutathione (GSH) acts as a key reducing agent and is oxidized to glutathione disulfide (GSSG) during ROS neutralization, while glutathione reductase (GR) regenerates GSH from GSSG using NADPH. The Fenton reaction is shown as a source of hydroxyl radicals (^•^OH).

The primary endogenous antioxidants include enzymes such as SOD, glutathione peroxidase (GPx), and CAT, which collectively counteract the harmful effects of ROS (Fig. 1). The functionality of these enzymes relies on the presence of trace elements like selenium, copper, manganese, and zinc, making their adequate dietary intake essential for cellular redox balance (Tainer et al. 1983, Frye et al. 2022). SOD catalyzes the conversion of $O_{2}^{•-}$ into H_2_O_2_, representing the first line of enzymatic defense against ROS (Fukai and Ushio-Fukai 2011). CAT subsequently decomposes H_2_O_2_ into H_2_O and O_2_, preventing its accumulation and oxidative damage (Nandi et al. 2019). GPx and glutathione S-transferases (GSTs) further contribute to antioxidant defense by reducing H_2_O_2_ and lipid hydroperoxides and by neutralizing secondary products of lipid peroxidation (Arthur 2000, Singhal et al. 2015).

### Oxidative Damage to Biomolecules (DNA, Lipids, and Proteins)

**Fig. 2.**
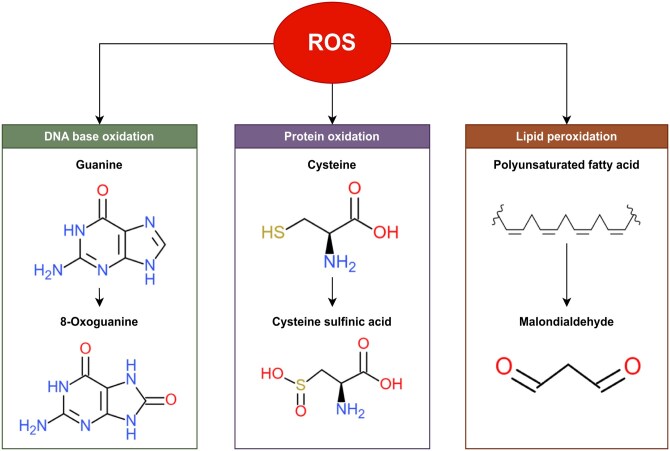
General schematic overview of reactive oxygen species (ROS)-induced oxidative damage to macromolecules. The diagram illustrates how ROS oxidize DNA bases, redox-sensitive amino acid residues in proteins, and polyunsaturated fatty acids in lipids. Examples include the formation of 8-oxoguanine in DNA, cysteine oxidation in proteins (eg formation of cysteine sulfinic acid), and lipid peroxidation products such as malondialdehyde (MDA).

Rather than remaining stable, free radicals tend to steal electrons from neighboring molecules, initiating oxidative damage to intracellular macromolecules, including DNA, proteins, and lipids (Fig. 2). In DNA, exposure to ROS leads to base modifications such as the conversion of guanine to 8-oxoguanine, which can mispair with cytosine or adenine, potentially resulting in mutations. These mutations may occur in both nuclear and mitochondrial DNA, contributing to double-strand breaks and genomic instability (Moskalev et al. 2013). Protein oxidation commonly affects amino acid side chains—particularly sulfur-containing residues like cysteine and methionine—altering protein structure and function and may also participate in ROS-dependent signaling pathways (Moskovitz and Oien 2010). ROS-induced lipid degradation, known as lipid peroxidation, targets polyunsaturated fatty acids within cellular membranes, generating secondary end-products such as MDA. Lipid peroxidation disrupts membrane architecture and function, compromising cellular integrity and homeostasis (Ayala et al. 2014). Various forms of oxidative damage—such as DNA lesions, lipid peroxidation, protein modifications, and mitochondrial alterations—tend to accumulate with age, implicating them as potential contributors to the aging process. Consequently, these oxidative biomarkers are being explored as indicators of biological age (Syslová et al. 2014).

### The Role of Oxidative Stress in Cellular and Tissue Aging

Oxidative stress, resulting from an imbalance between the production of ROS and the antioxidant defense system, plays a central role in aging and age-related diseases. With aging, the accumulation of ROS intensifies, disrupting normal physiological processes. The sources of oxidative stress are diverse, involving both external factors (eg environmental pollutants, diet, drugs, and radiation) and internal triggers (eg organelle dysfunction and increased activity of NADPH oxidase) (Yang et al. 2024a). These sources contribute to elevated ROS levels, resulting in sustained oxidative stress.

Historically, Hayflick and Moorhead (1961) introduced the concept of replicative senescence in mammalian cells, while Harman (2006) proposed the free radical theory of aging, postulating that the accumulation of oxidative damage induced by ROS is a major contributor to aging and age-associated functional decline. Numerous studies since then have confirmed that oxidative stress is a key driver of cellular senescence (Akshaj et al. 2016, Yang et al. 2024a, Stojanovic et al. 2025). Excessive ROS levels interfere with cell proliferation and promote senescence via multiple mechanisms, including mitochondrial dysfunction (Miwa et al. 2022), telomere shortening (Iskandar et al. 2025), DNA damage (Bujarrabal-Dueso et al. 2025), chronic inflammation (Baechle et al. 2023), lipid peroxidation (Ayala et al. 2014), and oxidative modification of proteins (Chakravarti and Chakravarti 2007). However, telomere shortening—one of the classical hallmarks of aging in mammals—is not associated with senescence in *D. melanogaster*, as flies maintain telomere length through retrotransposon-mediated mechanisms rather than telomerase activity. Together, these findings support the hypothesis that oxidative stress plays a pivotal role in the cellular aging process and is a major determinant of biological aging and age-related pathology.

Although ROS have traditionally been viewed as damaging agents driving cellular aging, recent evidence indicates that they also play signaling roles by reversibly modifying redox-sensitive cysteine residues. Such findings imply that maintaining redox balance, rather than complete ROS suppression, is essential for healthy aging (Lennicke and Cochemé 2020). Given its short lifespan and genetic tractability, *D. melanogaster* represents an ideal model to study these redox-regulated processes *in vivo*.

### *Drosophila melanogaster* as a Model Organism in Aging Research

*Drosophila* is a genus of small flies belonging to the order Diptera and the family Drosophilidae, commonly known as fruit or vinegar flies (Powell 1997). *D. melanogaster* is one of the most widely used animal models in genetics and gerontology, providing key insights into the molecular mechanisms of aging and longevity (Piper and Partridge 2018, Banerjee et al. 2019). Many age-related changes at the cellular and organismal level were first recorded in *Drosophila*, including the earliest identifications of genes with lethal phenotypic effects (Lints and Soliman 1988). This species serves as a powerful model for investigating the effects of various substances—such as antioxidants, phytochemicals, and dietary interventions—on aging and survival.

In *D. melanogaster*, as in other eukaryotes, oxidative stress is recognized as one of the main contributors to the aging process. Numerous studies have shown that increased ROS production during life correlates with progressive tissue dysfunction and shortened lifespan, while genetic manipulations that enhance the antioxidant defense system (eg via SOD and CAT) can lead to lifespan extension (Orr and Sohal 1994). In various organisms, including *Drosophila*, increased levels of protein carbonylation, lipid peroxidation, and glycation have been documented with age (Schwarze et al. 1998, Jacobson et al. 2010).

Studies using *D. melanogaster* have shown a strong correlation between antioxidant enzyme activity and lifespan. Suppression of endogenous antioxidant genes shortens lifespan, indicating their important protective role (Deepashree et al. 2022). Conversely, laboratory-evolved long-lived flies exhibit greater stress resistance and a more favorable antioxidant profile than control lines, with aging-related changes appearing later (Deepashree et al. 2019). Combined overexpression of antioxidant enzymes is also associated with increased longevity, while their deficiency or loss of function may result in elevated genomic instability (Orr and Sohal 1994). However, isolated overexpression of individual enzymes, such as CAT, does not necessarily lead to lifespan extension (Orr and Sohal 1992). Additionally, testing of various antioxidant drugs has shown that their effects on longevity depend on sex, dosage, and the timing of administration, highlighting the complex and context-dependent nature of ROS roles in aging processes (Magwere et al. 2006).

In addition to oxidative molecular damage, several other hallmarks of aging are present in *D. melanogaster* and ­contribute to the progressive decline in cellular function. Among these, disturbances in proteostasis—particularly protein aggregation—are strongly influenced by redox-sensitive signaling cascades such as the Forkhead box O (FOXO) transcription factor pathway, the p38 mitogen-activated protein kinase (p38 MAPK) signaling pathway, and the parkin-mediated pathway, which regulate cellular stress responses, autophagy, and protein turnover (Demontis and Perrimon 2010, Rana et al. 2013, Ryan et al. 2021). Recent advances in the biology of aging have identified several evolutionarily conserved signaling pathways that modulate lifespan across taxa. In *D. melanogaster*, 2 major nutrient-sensing networks—the insulin/insulin-like growth factor (IIS) and the Target of Rapamycin (TOR) pathways—play central roles in coordinating metabolism, stress responses, and longevity (Kenyon 2010, Partridge 2010, Partridge et al. 2011). Reduced IIS or TOR activity, achieved through genetic manipulation or pharmacological inhibition, has been shown to extend lifespan in flies, nematodes, and mammals, indicating a highly conserved mechanism of redox and metabolic regulation (Kapahi et al. 2004, Fontana et al. 2010). These pathways interact with downstream transcription factors such as FOXO, including its *Drosophila* homolog dFOXO, which promotes stress resistance and upregulation of antioxidant enzymes, including CAT and SOD. Additionally, stress-activated kinases such as c-Jun N-terminal kinase (JNK) can modulate dFOXO activity, linking external stress cues to endogenous antioxidant defenses (Hwangbo et al. 2004, Wong et al. 2008). Together, the IIS/TOR and JNK–FOXO networks integrate nutrient availability, oxidative stress, and metabolic state, forming a key regulatory axis that determines lifespan and functional decline during aging in *Drosophila*. ROS thus serve not only as damaging agents but also as second messengers that mediate redox-dependent post-translational modifications of key regulatory proteins, influencing lifespan and stress resistance.

Recent studies also highlight the crucial role of microbiota in shaping physiological traits associated with aging, including immunity, lifespan, and stress resilience (Arias-Rojas and Iatsenko 2022, Kristensen et al. 2024, Cho and Kang 2025). Notably, long-term exposure to lead(II) acetate—a common environmental pollutant—has been shown to significantly alter the composition and diversity of gut microbiota in *D. melanogaster*, suggesting an adaptive microbial response to chronic heavy metal stress (Beribaka et al. 2021a, 2021b). These changes are accompanied by increased microbial diversity across generations, which may contribute to the maintenance of host fitness under suboptimal conditions. Moreover, factors such as sex and population origin appear to modulate this response in a species-specific manner, indicating that host–microbiota interactions are intricately linked to environmental pressures and genetic background. Such findings provide a deeper understanding of the microbiota’s role as a mediator of oxidative stress and aging biomarkers in *Drosophila*.

Findings from *Drosophila* models are increasingly supported by clinical research linking oxidative stress markers with dietary and metabolic regulation in humans, underscoring the translational relevance of this model (Yu et al. 2016, Mazidi and Kengne 2019, Kanoni et al. 2021, Amanatidou et al. 2022). These findings suggest that dietary modulation of ROS pathways influences systemic health outcomes and reinforce the translational value of *Drosophila* models in identifying conserved mechanisms and reliable biomarkers of oxidative stress and aging.

*Drosophila* has emerged as a valuable model for studying cardiovascular diseases associated with aging. Evidence shows that cardiac tolerance to oxidative stress declines with age in *Drosophila*, primarily due to reduced levels of endogenous antioxidant enzymes such as SOD and CAT (Abete et al. 1999). This mirrors human aging, where oxidative stress plays a central role in the pathogenesis of age-related cardiovascular conditions, which remain the leading cause of mortality in elderly populations (Testa et al. 2010).

Despite the growing number of studies investigating oxidative stress and aging in *D. melanogaster*, the field remains highly heterogeneous with respect to selected biomarkers, physiological endpoints, and experimental approaches. A structured, descriptive, frequency-based synthesis is therefore needed to systematically map research trends, identify commonly used oxidative stress and aging markers, and highlight underexplored areas that warrant further investigation. The objectives of this review are to: (i) outline the key oxidative stress mechanisms relevant to antioxidant enzyme function and physiological aging; (ii) explain the rationale for using *D. melanogaster* as a model organism for oxidative stress and aging research; (iii) identify the most commonly used antioxidant enzyme-related biochemical markers and physiological aging indicators in *Drosophila* through systematic literature analysis; (iv) evaluate recent interventions affecting antioxidant enzyme activity and oxidative stress-related physiological outcomes in this model; and (v) discuss the broader translational relevance of *Drosophila*-based findings for aging and oxidative stress-related disease prevention.

In the context of this review, translational perspectives refer to the evolutionary conservation of oxidative stress pathways and antioxidant defense mechanisms between *D. melanogaster* and higher organisms, including mammals. Given the conserved roles of antioxidant enzymes, redox signaling, and key aging-related pathways, findings derived from *Drosophila* models can inform hypothesis-driven research on aging mechanisms, nutritional interventions, and redox-related disease prevention in more complex systems.

## Materials and Methods

This review employed a systematic approach to identify relevant scientific literature addressing the relationship between oxidative stress, antioxidant mechanisms, and aging in *D. melanogaster*. The literature search was designed to focus on studies explicitly reporting ROS, antioxidant enzyme activity, and physiological aging endpoints, ensuring high thematic specificity.

**Fig. 3.**
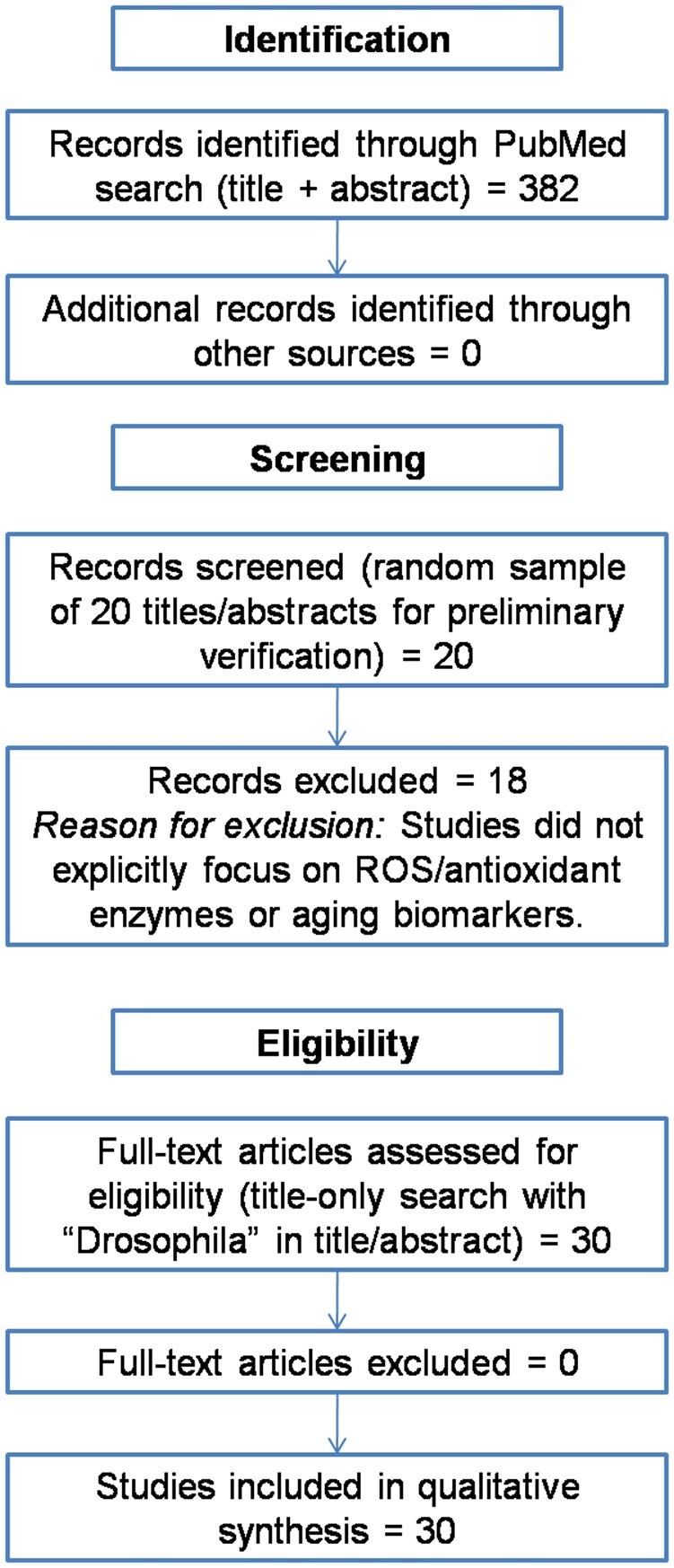
PRISMA flow diagram summarizing the literature search and study selection process for studies addressing oxidative stress, antioxidant markers, and aging biomarkers in *Drosophila melanogaster*.

To maintain focus and feasibility, the literature search was initially conducted using title and abstract fields for all key terms. A preliminary verification of this approach was performed using a random sample of 20 records retrieved through the title-and-abstract search. Of these, only 2 studies met the inclusion criteria for explicit measurements of ROS, antioxidant enzymes, and physiological aging indicators, while the remaining 18 were outside the defined focus of this review. Based on this assessment, the search strategy was refined by restricting all oxidative stress- and aging-related terms to the article title, while retaining *Drosophila* in the title or abstract. This refinement facilitated the identification of studies directly addressing oxidative stress, antioxidant enzymes, and aging biomarkers, without substantially expanding the dataset beyond core physiological aging indicators. Consequently, other relevant markers and pathways—such as protein carbonylation, protein aggregation, mitochondrial dysfunction, and upstream redox signaling—were not included, as they fell outside the predefined scope of this systematic review. The study selection process is summarized in the PRISMA flow diagram (Fig. 3). Study selection and data extraction were performed independently by 2 reviewers and finalized through consensus.

The literature search was carried out using the PubMed database (https://pubmed.ncbi.nlm.nih.gov/), one of the largest and most reliable sources of peer-reviewed biomedical publications. PubMed was selected because it provides comprehensive coverage of peer-reviewed biomedical and life science literature, including genetics, molecular biology, toxicology, and aging research, which are central to oxidative stress studies in *Drosophila*. The following search terms and Boolean operators were applied in the formulation of the search query:

(“oxidative”[Title] OR “reactive oxygen species”[Title] OR “ROS”[Title] OR “superoxide dismutase”[Title] OR “SOD”[Title] OR “catalase”[Title] OR “CAT”[Title] OR “antioxidation”[Title] OR “antioxidant”[Title]) AND (“aging”[Title] OR “ageing”[Title] OR “lifespan”[Title] OR “life-span”[Title] OR “life span”[Title] OR “longevity”[Title]) AND (“Drosophila”[Title/Abstract]).

Inclusion and exclusion criteria were defined a priori to ensure consistency, reproducibility, and relevance of the selected studies. Only peer-reviewed scientific articles meeting the following conditions were included:

- Inclusion criteria: (i) Studies that explicitly investigate the relationship between oxidative stress and aging in *D. melanogaster*; (ii) research that includes analysis of antioxidant enzymes such as SOD, CAT, and other oxidative stress markers; (iii) articles published between April 2020 and April 2025; (iv) publications written in English; and (v) experimental studies.
- Exclusion criteria: (i) Studies not focused on *D. melanogaster*; (ii) articles that do not address oxidative stress or aging; (iii) non-English publications; and (iv) review articles, meta-analyses, case reports, editorials, commentaries, and non-peer-reviewed materials.

The 5-year publication window was chosen to emphasize contemporary findings, reflecting recent methodological advances in oxidative stress measurement, genetic modulation, and experimental interventions. This time restriction also ensured alignment with updated experimental models and analytical techniques that have evolved substantially in recent years. A formal risk of bias or quality assessment was not performed, as the review aimed to provide a descriptive synthesis of reported oxidative stress markers and physiological aging parameters rather than a quantitative effect-size analysis.

### Data Extraction and Analysis

After selecting the relevant studies, a systematic content analysis was performed to summarize key findings linking oxidative stress, aging markers, and physiological effects in *D. melanogaster*. Data were extracted from 30 original research articles published in the last 5 years. For each study, the following parameters were recorded: research focus, ROS-related markers, aging-related biomarkers, main experimental results, and overall conclusions. The collected data were compiled into summary tables to facilitate comparative analysis and identification of consistent trends. Descriptive frequency analysis and thematic classification were performed without the use of specialized bibliometric software, based on predefined inclusion criteria and reported study characteristics.

#### Descriptive Frequency Analysis of ROS and Aging Markers

Identified ROS markers and aging indicators were quantified based on their frequency across the included studies. In parallel, the prevalence of various physiological aging indicators was analyzed. The results are presented in tabular format.

#### Thematic Classification of Studies

In addition to quantitative analysis, the studies were categorized thematically to better understand research priorities in the field. The reviewed studies were grouped into 6 main categories: (i) natural extracts and phytonutrients, (ii) genetic interventions and transgenic lines, (iii) signaling and metabolic pathways, (iv) environmental and nutritional stressors, (v) pharmacological agents, and (vi) observational studies without interventions. Classification was based on each study’s primary theme and methodological approach. The frequency of studies within each category is presented in tabular form.

## Results and Discussion

This review included 30 relevant studies published over the last 5 years that investigated the relationship between oxidative stress, physiological aging markers, and the effects of various interventions in *D. melanogaster*. The data enabled a detailed quantitative and thematic analysis of key parameters related to ROS markers, aging indicators, and main findings (Table 1).

**Table 1.** Summary of relevant studies on oxidative stress and aging in *Drosophila melanogaster*

Author (year)	Study topic	ROS marker	Aging marker	Main findings
**Zhu et al. (2025)**	*Poria cocos* polysaccharide (PCP) delays aging by enhancing antioxidant capacity and suppressing the expression of branched-chain amino acid transferase genes in *D. melanogaster*	MDA, antioxidant enzyme activity (measured levels of SOD and CAT)	Lifespan, locomotor activity	PCP supplementation extended lifespan, improved locomotor performance, alleviated intestinal dysfunction, increased SOD and CAT activities, reduced MDA levels, and downregulated branched-chain amino acid transferase gene expression.
**Sun et al. (2025)**	Effect of apigenin on oxidative stress resistance and proteostasis via the PTEN-AKT signaling pathway	ROS levels, antioxidant enzyme activity	Lifespan, survival rate	Apigenin extends lifespan, reduces ROS, increases antioxidant activity, activates the proteostasis network, regulates PTEN, decreases p-AKT, and affects FOXO/S6K signaling.
**Santos et al. (2024)**	Effect of *Pereskia aculeata* supplementation on longevity and antioxidant status in *D. melanogaster*	ROS levels, MDA, antioxidant enzyme activity (measured SOD and CAT levels)	Lifespan, survival rate, locomotor activity	10% *Pereskia aculeata* supplementation reduces ROS and increases longevity, while all concentrations decrease CAT activity and lipid peroxidation. AChE activity was reduced in all groups.
**Quintero-Espinosa et al. (2024)**	Effect of the LRRK2 kinase inhibitor PF-06447475 on paraquat-induced neurodegeneration in *D. melanogaster*	MDA, antioxidant status	Lifespan, locomotor activity, neurodegeneration	PF-06447475 reduces paraquat-induced oxidative stress, improves motor function, and extends lifespan.
**Feng et al. (2024)**	Study on the characterization of polysaccharides from *Bupleurum chinense* (BCPRs) focusing on antioxidant mechanisms of ROS-related signaling pathways and evaluation of antiaging effects in *D. melanogaster* model	Antioxidant enzyme activity (measured levels of SOD, GPx), MDA	Lifespan	BCPR-3 demonstrated strong in vitro radical scavenging capacity (DPPH, ABTS, OH, O_2_-), enhanced antioxidant enzyme activity, and reduced MDA in cells. In *in vivo* experiments, BCPR-3 extended lifespan.
**Yang et al. (2024b)**	Antioxidant and geroprotective effects of *Artemisia argyi* extract in *D. melanogaster*	Antioxidant enzyme activity (measured levels of SOD1, SOD2, and CAT), MDA, antioxidant gene expression (mRNA levels): SOD1, SOD2, and CAT	Lifespan, locomotor activity	*Artemisia argyi* extract extended fly lifespan, improved motor function and tolerance to H_2_O_2_, increased antioxidant enzyme activities and expression of SOD1, SOD2, and CAT, while reducing MDA levels.
**Golubev et al. (2024)**	Effect of *Berberis vulgaris* extract on lifespan and health of *Drosophila* through sex-dependent antioxidant activity	Antioxidant activity (measured in erythrocytes—*in vitro*), antiglycation activity	Lifespan, stress resistance, locomotor activity, intestinal barrier integrity	*Berberis vulgaris* extract led to lifespan extension, increased stress resistance, and improved locomotor activity.
**Liu et al. (2023)**	Polysaccharide from *Agrocybe aegerita* (AAPS) combined with *Bifidobacterium lactis* Bb-12 alleviates aging-related oxidative stress and restores gut flora in *D. melanogaster*	Antioxidant enzyme activities (measured levels of T-SOD, CAT, GPx), MDA	Lifespan, locomotor activity	AAPS and the Bb-12 complex significantly increased lifespan in male and female *D. melanogaster*.
**Tuo et al. (2023)**	Effect of *Angelica sinensis* polysaccharide (ASP) on lifespan and aging-related diseases via insulin and TOR signaling pathways and antioxidant capacity in *Drosophila*	Inhibition of IIS and TOR signaling, antioxidant capacity	Lifespan, locomotor activity, reproduction, resistance to starvation and oxidative stress, balanced gut homeostasis, improved sleep, neuroprotection	ASP treatment extended lifespan and reproduction, improved locomotor performance and stress resistance, preserved gut homeostasis, alleviated sleep disturbances, and exerted neuroprotective effects in aged flies.
**Yang et al. (2023)**	Antioxidant effects of *Hedysarum polybotrys* polysaccharide (HPS) on lifespan extension and aging-related diseases in *D. melanogaster*	Antioxidant status	Lifespan, eclosion rate, balanced gut homeostasis, improved sleep, neuroprotection (AD model)	HPS led to an increased eclosion rate and extended lifespan, as well as alleviation of aging symptoms such as imbalanced gut homeostasis and sleep disturbances.
**Yan et al. (2022)**	Quercetin prevents aging of intestinal stem cells (ISC) by removing ROS and inhibiting insulin signaling in *Drosophila*	ROS level, expression analysis of antioxidant genes *Cat*, *Sod1*, *Sod2*, and *GstD1*	Lifespan, ISC hyperproliferation, intestinal homeostasis	Quercetin supplementation can prevent ISC aging, maintain intestinal homeostasis, and extend lifespan in *Drosophila*. Additionally, quercetin can accelerate intestinal damage recovery and improve tolerance to stress stimuli.
**Liu et al. (2022)**	Antiaging effect of *Agrocybe aegerita* polysaccharide (AAPS) through regulation of oxidative stress and gut microbiota in *D. melanogaster*	Antioxidant enzyme activity (measured levels of T-SOD, CAT, and GPx), MDA	Lifespan	AAPS extends lifespan and eliminates H_2_O_2_-induced oxidative stress. AAPS reshaped the disrupted gut microbiota and increased the abundance of beneficial *Lactobacillus* bacteria.
**Feng et al. (2022)**	Effect of Se-enriched *Chrysanthemum morifolium* (SeCM) on lifespan and expression of antioxidant genes in *Drosophila*	MDA, expression analysis of antioxidant genes Cu/Zn-SOD, Mn-SOD, and CAT	Lifespan	SeCM reduces MDA levels, increases the activity of endogenous antioxidant enzymes and the expression of genes responsible for antioxidant defense, thereby extending the lifespan of fruit flies.
**Baek et al. (2022)**	Role of the dual oxidase enzyme, which produces hydrogen peroxide, in neuronal oxidative damage and lifespan in *D. melanogaster*	ROS level, expression analysis of antioxidant genes *Cat, Sod1*, and *Sod2*	Lifespan, oxidative stress resistance, locomotor activity, neurodegeneration	Heterozygous *duox* mutants’ exhibit extended lifespan, while neuronal *duox* knockdown reduces oxidative stress, indicating a neuronal role in aging.
**Zhang et al. (2022)**	Antioxidant effect of aqueous extract of *Astragalus membranaceus* (ARE) on aging in *D. melanogaster*	Antioxidant enzyme activity (measured levels of SOD and CAT)	Lifespan, locomotor activity, glutamate level	ARE significantly extends lifespan, improves motor function and food intake, lowers glutamate levels, and increases antioxidant activity (SOD, CAT). It also exhibits free radical scavenging capacity.
**Malacrida et al. (2022)**	Effect of 24-h hypoxia on lifespan and ROS levels in different *D. melanogaster* strains	ROS levels	Lifespan, survival rate	Sod1^n1^ flies exhibit the highest ROS levels and the shortest lifespan. Canton-S shows the lowest ROS levels and the lowest mortality. Hypoxia amplifies these differences, and genetic background significantly influences ROS levels and survival after hypoxia.
**Graham et al. (2022)**	Mitochondrial ROS signaling via reverse electron transport (RET) and its loss during aging in *D. melanogaster*	mtROS levels	Lifespan, electron flow through the electron transport chain (ETC)	ROS-RET arises from increased electron entry into the ETC and requires functional mitochondria. With aging, ROS-RET is lost and replaced by chronically elevated mtROS levels. In aged individuals, ROS-RET is absent, and mtROS remains constantly high.
**Musachio et al. (2022)**	Sex-specific changes in oxidative stress parameters and longevity induced by Bisphenol F and S compared to Bisphenol A in *D. melanogaster*	MDA, antioxidant enzyme activity (measured levels of SOD and CAT)	Lifespan (in females)	Toxicological effects vary between sexes; BPA is more harmful than BPF and BPS in male flies.
**John et al. (2022)**	Effect of Jobelyn supplementation on longevity, motor functions, and antioxidant status in *D. melanogaster* exposed to lipopolysaccharide (LPS)	Hydrogen peroxide and nitric oxide accumulation, antioxidant status	Lifespan, mortality rate, locomotor activity	Jobelyn reduces mortality and oxidative stress, improves motor functions, decreases acetylcholinesterase activity, and extends lifespan.
**Golubev et al. (2022)**	Antioxidant effects and aging delay by *Lonicera pallasii* extract (LE) and cyanidin-3-O-glucoside (C3G) in *D. melanogaster*	Radical scavenging, oxidative hemolysis of erythrocytes	Lifespan, stress resistance, locomotor activity, intestinal barrier integrity	LE and C3G increase lifespan, enhance stress resistance, reduce locomotor activity in females, and improve intestinal barrier integrity.
**Chen et al. (2021)**	*Bifidobacterium adolescentis* regulates CAT activity and host metabolism and improves healthspan across multiple species	Antioxidant enzyme activity (measured CAT levels)	Healthspan and overall lifespan	Dietary supplementation with *B. adolescentis* improved osteoporosis and neurodegeneration in mice and increased healthspan and lifespan in *D. melanogaster* and *C. elegans*. Supplementation also increased CAT enzyme activity.
**Deepashree et al. (2022)**	Genetic repression of antioxidant enzymes shortens lifespan in *D. melanogaster*	Repression of antioxidant enzymes (measured levels of SOD1 and CAT)	Lifespan	Genetic repression of SOD1 and CAT in transgenic flies led to a drastic reduction in longevity.
**Sheng et al. (2021)**	Effect of caffeic acid (CA) on aging of intestinal stem cells (ISC) in *Drosophila*	ROS levels, gene expression related to oxidative resistance (including *SOD*, *Cat*, *gclc*, and *gstD1*), antioxidant enzyme activity (measured levels of SOD and CAT), MDA	ISC hyperproliferation, loss of intestinal function, survival rate	CA reduces oxidative stress, inhibits JNK signaling, prevents ISC hyperproliferation, improves intestinal function and stress survival.
**Cheng et al. (2021)**	Protective effects of an antioxidant mixture of curcumin and broccoli seed extract on neurodegeneration and longevity in *Drosophila*	Indirect: oxidative stress induced by paraquat	Lifespan, neurodegeneration of dopaminergic (DA) neurons	The mixture extends lifespan (both components individually and combined), induces changes in the expression of over 70 genes, protects against DA neurodegeneration, and increases resistance to paraquat.
**Yang et al. (2021)**	Extension of *Drosophila* lifespan by *Astragalus* polysaccharide through an antioxidant- and insulin/IGF-1 signaling-dependent mechanism	Antioxidant enzyme activity (measured levels of SOD and CAT), resistance to H_2_O_2_	Lifespan, locomotor activity, starvation resistance, reproduction	*Astragalus* polysaccharide extends lifespan, improves mobility, reproduction, and stress resistance, and also increases the expression of antioxidant genes.
**Ma et al. (2021)**	Effect of carrageenan oligosaccharide on lifespan and healthspan extension in male *D. melanogaster*	Antioxidant enzyme activity (measured levels of Cu, Zn-SOD and CAT), MDA	Lifespan, locomotor activity, fecundity	Carrageenan oligosaccharide extends lifespan, improves mobility and fecundity. Additionally, it increases antioxidant enzymes, reduces MDA, and enhances microbiota diversity.
**Wen et al. (2020)**	Endurance exercise alleviates the negative effects of a high-salt diet (HSD) in *Drosophila*, including reduced motor ability and shortened lifespan, through enhanced antioxidant activity	Antioxidant enzyme activity (measured levels of SOD), MDA	Lifespan, locomotor activity	HSD decreases lifespan, climbing ability, and SOD levels, while increasing MDA levels. Exercise and dFOXO improve all these parameters.
**Koliada et al. (2020)**	Effect of mating status on lifespan, metabolism, and antioxidant system in *Drosophila*	Antioxidant enzyme activity (measured levels of SOD and PX), content of advanced glycation end products (AGE)	Lifespan, fat amount, number and quality of eggs, expression of InR and dTOR	Virgin females showed the longest lifespan, while polyandrous females had the shortest; mating reduced food intake and fat reserves but increased egg number and quality. Mating also altered SOD, PX, and AGE levels, with increased InR and decreased dTOR expression in polyandrous females.
**Zhu et al. (2020)**	Antioxidant and geroprotective activity of polysaccharides from *Cordyceps cicadae*	Antioxidant enzyme activity (measured levels of CAT and GPx), MDA, expression analysis of oxidation resistance-related genes (*CAT, SOD1, MTH*)	Lifespan	The CP70 fraction of polysaccharides from *Cordyceps cicadae* significantly extends lifespan, increases CAT and GPx activities, reduces MDA levels, and upregulates expression of CAT, SOD1, and MTH genes.
**Yamakawa-Kobayashi et al. (2020)**	Loss of the carnosinase gene (CNDP) causes shortened lifespan and increased sensitivity to oxidative stress in *D. melanogaster*	GSH and H_2_O_2_ as indirect indicators of ROS	Lifespan	*Dcndp* mutant flies show reduced lifespan and increased sensitivity to oxidative stress induced by paraquat or H_2_O_2_.

*Abbreviations*: ROS, reactive oxygen species; mtROS, mitochondrial reactive oxygen species; SOD, superoxide dismutase; T-SOD, total superoxide dismutase; CAT, catalase; MDA, malondialdehyde; GPx, glutathione peroxidase; PX, peroxidase; IIS, insulin/insulin-like growth factor signaling pathway; TOR, target of rapamycin signaling pathway; ISC, intestinal stem cells; AD, Alzheimer’s disease; MTH, methuselah gene.

**Fig. 4.**
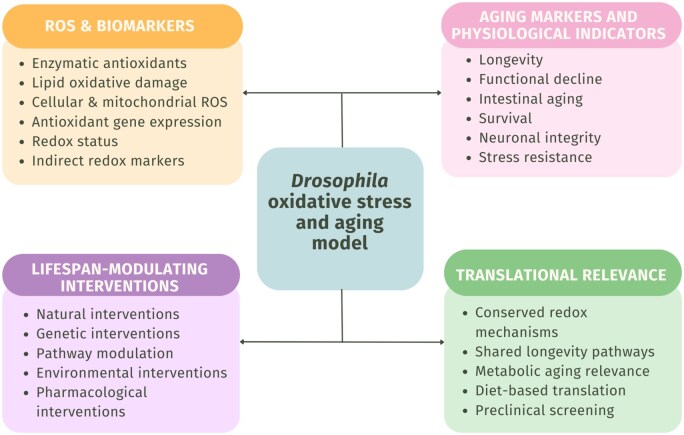
Schematic representation of oxidative stress-related biomarkers, physiological aging indicators, lifespan-modulating interventions, and translational relevance in *Drosophila melanogaster*. The diagram highlights antioxidant defense markers, functional aging outcomes, major intervention categories, and conserved pathways relevant to aging research.

To provide an integrative overview, a conceptual diagram summarizing the key interactions between oxidative stress, aging biomarkers, physiological decline, and interventions is presented in Fig. 4. The schematic overview integrates findings from the reviewed studies and visually summarizes how antioxidant defense systems interact with physiological aging outcomes in *D. melanogaster*. This framework provides the context for the subsequent descriptive frequency analysis of ROS markers and lifespan-related parameters. Importantly, several of the summarized mechanisms and intervention outcomes reflect conserved oxidative stress and aging pathways, providing a conceptual basis for the translational relevance discussed in later sections.

Quantitative analysis revealed the frequency of antioxidant enzymes used (SOD, CAT, and GPx) and indirect oxidative damage markers such as MDA (Table 2), as well as variations in physiological aging parameters, including lifespan and locomotor activity (Table 3).

**Table 2.** Markers of oxidative stress in *Drosophila melanogaster*

Type of ROS marker	Number of studies	Examples
**Antioxidant enzyme activity**	17	Feng et al. (2024), Yang et al. (2024a, 2024b), Wen et al. (2020)
**Lipid peroxidation (malondialdehyde, MDA)**	13	Zhu et al. (2025), Liu et al. (2022), Ma et al. (2021)
**Total reactive oxygen species (ROS)/mitochondrial reactive oxygen species (mtROS) levels**	7	Sun et al. (2025), Santos et al. (2024)
**Expression of antioxidant genes**	6	Feng et al. (2022), Zhu et al. (2020), Sheng et al. (2021)
**Antioxidant status**	3	Quintero-Espinosa et al. (2024), Yang et al. (2023), John et al. (2022)
**Glutathione (GSH) or H_2_O_2_ as indirect indicators**	2	Yamakawa-Kobayashi et al. (2020), John et al. (2022)

**Table 3.** Aging markers and physiological indicators

Type of aging/physiological effect marker	Number of studies	Examples
**Lifespan**	29	All studies except Sheng et al. (2021)
**Locomotor activity**	14	Yang et al. (2024b), Liu et al. (2023), Zhang et al. (2022)
**Gut homeostasis/ISC aging**	6	Golubev et al. (2024), Tuo et al. (2023), Yan et al. (2022)
**Survival/mortality rate**	5	Sun et al. (2025), Santos et al. (2024)
**Neuroprotection/neurodegeneration**	5	Quintero-Espinosa et al. (2024), Cheng et al. (2021)
**Oxidative stress resistance**	4	Tuo et al. (2023), Golubev et al. (2024)

Thematic classification of the studies revealed dominant research approaches and priorities in the field, with clear emphasis on natural extracts, signaling and metabolic pathways, and pharmacological agents (Table 4).

**Table 4.** Thematic classification of studies on *Drosophila melanogaster* (oxidative stress and aging)

Category	No. of studies
**Natural extracts/phytochemicals**	21
**Genetic interventions/transgenic lines**	3
**Signaling and metabolic pathways**	6
**Environmental/nutritional stressors**	3
**Pharmacological agents/drugs**	5
**Functional analysis without intervention**	2

### ROS and Antioxidant Defense Enzymes

The findings of this systematic review demonstrate a strong association between oxidative stress and aging-related physiological changes in *D. melanogaster*. Antioxidant enzymes such as SOD and CAT were among the most frequently used markers of oxidative stress, underlining their central role in maintaining redox homeostasis. More than half of the analyzed studies included enzymatic antioxidants such as SOD, CAT, and GPx, emphasizing their importance in modulating oxidative damage and supporting physiological stability. Upregulation of these enzymes is typically associated with increased lifespan and enhanced stress resistance, whereas genetic repression or pharmacological inhibition leads to reduced survival and accelerated aging. The central role of these enzymes in lifespan regulation highlights the evolutionary conservation of redox defense mechanisms, which underpins the relevance of *Drosophila* as a translational model for oxidative stress-associated aging processes.

In *Drosophila*, these antioxidant defense systems act in a coordinated manner. SOD catalyzes the conversion of $O_{2}^{•-}$ into H_2_O_2_, which is then degraded by CAT, preventing the formation of highly reactive hydroxyl radicals (Hong et al. 2024). Another key component of the redox defense is the thioredoxin reductase (TrxR) system, which maintains thioredoxin in its reduced state and contributes to glutathione (GSH) redox homeostasis in the absence of a canonical glutathione reductase gene in flies. Mutations in the gene *dmtrxr-1*, encoding TrxR, impair redox balance and lead to developmental arrest, increased pupal mortality, and drastically shortened adult lifespan (Missirlis et al. 2001). Functional interactions between TrxR and the SOD/CAT system suggest that these enzymatic pathways share the burden of ROS detoxification. Combined deficiencies exhibit synergistic effects, with more severe phenotypes than single mutants. Overexpression studies reveal that CAT can partially rescue the effects of TrxR deficiency by reducing H_2_O_2_ accumulation, while excess SOD activity without adequate CAT function exacerbates oxidative stress. These findings confirm that an integrated antioxidant network is essential for stress resistance, developmental progression, and lifespan regulation in *Drosophila* (Missirlis et al. 2001, Orr et al. 2013, Deepashree et al. 2022).

### Lipid Peroxidation as an Indirect Marker

Indirect indicators like MDA were widely applied to assess lipid peroxidation and oxidative damage caused by ROS. MDA was reported in 13 studies, confirming its role as a reliable marker for oxidative damage in aging processes. Elevated MDA levels are generally associated with cellular membrane damage, reduced locomotor activity, and shortened lifespan. However, several interventions that reduce MDA—such as treatment with plant polysaccharides or probiotic supplementation—also concurrently upregulate endogenous antioxidant enzymes, suggesting a coupled and possibly synergistic effect.

Moreover, several studies in *Drosophila* link MDA elevations directly to functional decline associated with aging. For example, supplementation with dietary polyunsaturated fatty acids such as MAG-EPA not only increased lifespan by ∼15% but also significantly reduced lipid peroxidation in thoracic muscle, as indicated by lower MDA levels, while preserving SOD activity (MAG-EPA delayed lipid peroxidation and maintained antioxidant enzyme function) (Champigny et al. 2018). Similar results were reported with *Agrocybe aegerita* polysaccharides: treated flies showed both lifespan extension and decreased MDA, along with increased activity of endogenous antioxidants (Liu et al. 2022). The antioxidant effect of chitosan, a natural polysaccharide derived from chitin, was demonstrated in *D. melanogaster* females, where chitosan supplementation decreased ROS and MDA levels while increasing SOD and CAT activities (Güneş and Nizamlıoğlu 2023). These findings support the role of natural polymers as potent modulators of oxidative stress, particularly in reducing lipid peroxidation and enhancing enzymatic antioxidant defenses. Curcumin supplementation also upregulated SOD activity and lowered MDA accumulation—contributing to prolonged survival (Shen et al. 2013). Olive-derived phenolic compounds have been shown to exert both antioxidant and pro-oxidant effects in *D. melanogaster*, depending on concentration and dietary context. Supplementation with olive leaf and fruit extracts altered developmental parameters, survival, and lifespan, while modulating MDA levels and GST activity (Güneş and Danacıoğlu 2018). Phytosterols extracted from mountain-cultivated ginseng (MCG) have recently been identified as potent regulators of aging and oxidative stress in *D. melanogaster*. Compared with cultivated ginseng (CG), MCG contains higher concentrations of stigmasterol, a compound with strong structural similarity to steroid hormones (Liu et al. 2024). Supplementation with MCG- and CG-derived phytosterols extended the natural lifespan of flies and improved multiple parameters of healthy aging, including progeny number, sleep duration, climbing performance, and survival under oxidative stress. In a Parkinson’s disease fly model (*parkin* mutant), resveratrol reduced MDA and H_2_O_2_ levels while improving climbing performance, confirming its powerful antioxidant and neuroprotective effects (Adedara et al. 2022). These studies collectively show that interventions that reduce MDA not only preserve membrane integrity but are also associated with measurable improvements in stress tolerance and lifespan. Such conserved relationships between lipid peroxidation, antioxidant defenses, and functional aging outcomes provide a foundation for considering broader translational implications.

In a more recent study, time-restricted feeding was shown to markedly decrease MDA concentrations and enhance CAT activity in the brains of aged *D. melanogaster*, thereby reducing oxidative stress and improving locomotor performance (Abdulwahab et al. 2025). This finding further supports the role of dietary and metabolic interventions in mitigating lipid peroxidation and promoting neuronal resilience during aging.

Furthermore, evidence suggests that the reduction of MDA and lipid peroxidation is mediated via well-known stress–response pathways. Resveratrol’s life-extending effects are dependent on FOXO activation, which involves downstream upregulation of CAT and other antioxidants, thereby suppressing oxidative stress and lipid peroxidation (Xiang et al. 2011). Activation of JNK–FOXO signaling also induces antioxidant peroxiredoxins—such as Jafrac1—mitigating neuronal oxidative damage and reducing MDA-related lipid peroxidation in the brain (Lee et al. 2009). Moreover, probiotic and synbiotic supplementation—shown to diminish total oxidant burden and lipid peroxidation levels—suggests possible modulation of gut–brain redox communication, indicating that reduced MDA may reflect systemic benefits extending beyond the gut (Westfall et al. 2018).

However, it is important to note that not all antioxidant or phytochemical interventions consistently produce beneficial outcomes. For example, the effects of resveratrol on lifespan and oxidative protection in *Drosophila* remain inconsistent across studies. While some experiments have reported clear antioxidative and neuroprotective effects, others found little or no lifespan extension in wild-type strains, or even neutral outcomes depending on dosage, genetic background, and diet composition (Staats et al. 2018, Adedara et al. 2022, Lushchak et al. 2023). These discrepancies highlight the complexity of redox balance and suggest that resveratrol’s effects may depend on specific experimental conditions rather than representing a universally beneficial antioxidant.

Altogether, these findings support a model in which lowering lipid peroxidation (measured by MDA) activates integrated redox and stress–response networks that delay age-related physiological decline in *Drosophila*.

### Physiological Aging Markers

Lifespan was the most commonly assessed aging marker, analyzed in nearly all reviewed studies, followed by locomotor activity, gut/stem cell aging, and survival rate. These physiological parameters collectively reflect the functional state of the organism and its capacity to resist age-associated decline.

#### Lifespan and Functional Performance

Lifespan remains the most frequently utilized physiological marker in *Drosophila* aging studies due to its simplicity, reproducibility, and direct relevance to organismal aging. Numerous interventions—genetic, dietary, or pharmacological—have been evaluated primarily through their effect on median or maximum lifespan, making it a central metric in aging research (Tower 2000, Partridge et al. 2005, Feng et al. 2022). However, lifespan alone does not capture the full spectrum of aging-related decline. For this reason, complementary markers such as locomotor activity and stress resistance are often included to provide a more comprehensive functional assessment (Golubev et al. 2024, Zhu et al. 2025). For example, age-associated reductions in climbing ability (negative geotaxis) are commonly used to assess neuromuscular function, and these declines typically precede mortality in flies (Gargano et al. 2005). Interventions that extend lifespan often simultaneously preserve locomotor performance, indicating that physiological healthspan and longevity are tightly coupled. The coupling of lifespan extension with preserved functional performance supports the use of *Drosophila* as a model for evaluating interventions relevant to healthspan, a concept central to translational aging research.

#### Tissue-Specific Aging and Gut Homeostasis

In addition to behavioral and survival parameters, markers of tissue-specific aging—such as gut integrity and intestinal stem cell (ISC) function—are increasingly used as sensitive indicators of systemic aging. Age-related dysregulation of gut homeostasis, including ISC hyperproliferation and epithelial barrier dysfunction, has been linked to systemic inflammation and reduced survival (Sheng et al. 2021, Golubev et al. 2022, Yan et al. 2022). Maintenance of gut function, either through genetic manipulation (eg FOXO activation and JNK suppression) or dietary interventions (eg probiotics and antioxidants), not only preserves intestinal architecture but also delays the onset of age-related pathologies and improves overall survival (Zhou et al. 2011, Lian et al. 2018, Westfall et al. 2018, Sheng et al. 2021). These findings underscore the utility of combining multiple physiological markers—including lifespan, locomotion, gut homeostasis, and stress resistance—to evaluate the efficacy of antiaging interventions in *Drosophila*.

### Effects of Phytochemicals

#### General Mechanisms and Representative Compounds

Phytochemical interventions dominate the current literature, with 21 out of 30 studies employing natural extracts such as flavonoids, polysaccharides, and polyphenols. These compounds often exhibit dual functions: direct radical scavenging and indirect modulation of genetic pathways involved in stress response (eg FOXO, TOR, and PTEN/AKT). Although generally beneficial, effects are often dose-dependent and sex-specific, pointing to a need for individualized approaches in nutraceutical research.

Phytochemicals, particularly flavonoids, polyphenols, and plant-derived polysaccharides, are prominent in *Drosophila* aging research due to their reported antioxidant, anti-inflammatory, and lifespan-extending properties. Many of these compounds function both as direct ROS scavengers and as modulators of genetic pathways associated with aging and cellular stress responses. For instance, quercetin and curcumin have been shown to activate FOXO-dependent transcription and enhance SOD and CAT expression, leading to improved oxidative stress resistance and increased lifespan (Xiang et al. 2011, Subramanian et al. 2017, Fernandes et al. 2023). Polysaccharides from *Lycium barbarum* and *Acanthopanax senticosus* similarly improve mitochondrial function and upregulate antioxidant enzyme activity, often through modulation of the Notch and TOR pathways (Tang et al. 2019, Zhang et al. 2020). These findings support a dual mechanism of action, where phytochemicals act both at the biochemical level (neutralizing ROS) and at the genetic level (modulating stress signaling).

#### Dose-, Sex-, and Context-Dependent Effects

Phytochemical efficacy is frequently dose-dependent and influenced by sex, developmental stage, and baseline physiological condition. For example, resveratrol extends lifespan at low to moderate doses but exhibits toxic effects at higher concentrations or when administered chronically (Baur et al. 2006). Likewise, studies on green tea polyphenols have shown significant sex-specific effects on survival, activity, and antioxidant capacity, with males often showing greater responsiveness; catechins from Longjing green tea significantly increased SOD and CAT activity and gene expression in *D. melanogaster*, suggesting that lifespan extension by green tea catechins is mediated, at least in part, through upregulation of endogenous antioxidant defenses (Li et al. 2007, Lopez et al. 2014). These observations highlight the importance of context in nutraceutical interventions and underscore the need for precision in dosing and timing. Future studies should adopt stratified experimental designs to account for sex- and dose-dependent responses, potentially enabling the development of individualized phytochemical-based strategies for promoting healthy aging. These context-dependent responses underscore the importance of model organisms such as *Drosophila* for preclinical screening and mechanistic evaluation prior to translational consideration in more complex systems.

Although the majority of studies report positive effects of phytochemicals on lifespan and oxidative balance, some inconsistencies remain, particularly with compounds such as resveratrol and quercetin. These agents can act as either antioxidants or pro-oxidants depending on concentration and physiological context, indicating the need for more standardized experimental designs and inter-laboratory validation.

### Translational Relevance

Although *Drosophila* is a simple model organism, many oxidative stress-related pathways are evolutionarily conserved (Garofalo 2002, Bharucha 2009). Given the evolutionary conservation of oxidative stress mechanisms across species, insights gained from these studies may guide the development of antioxidant strategies relevant to aging-related processes and disease prevention in humans (Kanoni et al. 2021, Amanatidou et al. 2022). Interventions that extend lifespan and improve resilience in flies—especially those targeting insulin/IGF-1 signaling, mitochondrial function, or gut integrity—hold promise for translation into higher organisms, including humans (Ruetenik and Barrientos 2015, Westfall et al. 2018, Ma et al. 2020, Lee et al. 2023). Probiotic and phytochemical treatments are especially attractive due to their relative safety and accessibility. However, interspecies differences must be acknowledged when extrapolating findings.

Supporting this translational relevance, recent translatomic profiling of *Drosophila* oenocytes has revealed that aging and oxidative stress significantly alter the expression of genes involved in ROS metabolism. Key antioxidant enzymes such as SOD1, CAT, and peroxiredoxin 5 are downregulated in aged flies, mirroring molecular changes observed in mammalian liver aging (Huang et al. 2019). These findings highlight oenocytes as a promising tissue for investigating conserved redox mechanisms and systemic physiological deterioration associated with aging.

Evidence from *Drosophila* models has already informed early-stage human studies, particularly in the context of metabolic and hepatic aging. For instance, dietary antioxidants and probiotics have been shown to mitigate oxidative damage and improve redox balance in flies and in early-stage human studies (Moraes and Montagne 2021, Lopez-Ortiz et al. 2023). The robustness and scalability of *Drosophila* make it a powerful platform for preclinical screening, personalized nutrition research, and antioxidant therapy development. Bridging the gap between fly-based findings and clinical applications remains a key objective for future translational research.

Taken together, while the reviewed evidence supports a conserved role of oxidative stress in aging, contradictory findings—particularly for phytochemical interventions—underscore the need for cautious interpretation.

### Limitations

This review highlights several inherent limitations that should be considered when interpreting the findings. One major limitation lies in the variability of methodological approaches and the relatively small number of studies that simultaneously assessed multiple biochemical and physiological markers. The heterogeneity in experimental designs—such as differences in fly strain, age, sex, diet composition, and treatment duration—makes direct comparisons challenging and may contribute to inconsistencies among reported outcomes.

Furthermore, the literature search strategy was limited to studies published between April 2020 and April 2025, which ensured the inclusion of recent advances but also excluded older progressive studies that collectively explored related aspects of interventions (eg lifespan, oxidative biomarkers, and antioxidant gene expression across different publications). Consequently, earlier but mechanistically relevant studies may not have been captured in this systematic selection, representing a temporal limitation.

Another limitation concerns the restriction of search terms to article titles, which likely excluded studies where key terms such as oxidative stress, antioxidant response, or aging were present only in abstracts. Similarly, studies focused on upstream regulatory pathways—including FOXO, p38 MAPK, and Nrf2/Keap-1—were not systematically included, as the current analysis primarily emphasized downstream enzymatic and biochemical outcomes. In addition, other relevant markers and pathways, including protein carbonyls, protein aggregation, and mitochondrial dysfunction, were not analyzed in this review. While these pathways were conceptually discussed in the introduction, their exclusion from quantitative synthesis limits the integrative interpretation of signaling-level regulation in aging.

In addition to biochemical markers, physiological indicators such as lifespan and locomotor activity were variably reported across studies. Lifespan remains the most direct phenotype for assessing aging, while climbing ability (negative geotaxis) is commonly used as a proxy for neuromuscular function and vitality in aging *Drosophila* (Gargano et al. 2005, Rera et al. 2012). However, inconsistencies in experimental protocols—including differences in assay design, timing of behavioral testing, and sample size—reduce data comparability and complicate meta-analytical interpretation.

Thematic analysis of the included studies further revealed significant variation in methodological quality, measured parameters, and reporting standards. The effects of different interventions were found to be highly context-dependent, influenced by age, sex, genetic background, and specific experimental conditions. Some compounds that display protective effects in one genotype or context may exhibit neutral or even adverse outcomes in another, underscoring the importance of model characterization and experimental reproducibility.

Additionally, relatively few studies simultaneously assessed multiple markers, limiting the integrative understanding of the relationship between biochemical parameters of oxidative stress and complex physiological phenotypes. Moreover, the evidence base is dominated by studies reporting positive or lifespan-extending effects, whereas contradictory or null findings are comparatively underrepresented, which may introduce publication bias. Finally, environmental factors—such as exposure to heavy metals, temperature variation, or microbiota composition—remain insufficiently controlled or reported, further limiting cross-study comparability.

## Conclusions

This review confirms a strong link between oxidative stress and physiological aging in *D. melanogaster*, with antioxidant enzymes SOD and CAT as primary ROS markers. Lifespan and locomotor activity were the most used aging indicators, while natural extracts emerged as the most frequent interventions. Genetic and molecular studies further emphasized the model’s utility in exploring intrinsic defense mechanisms.

Collectively, these results highlight the value of *D. melanogaster* as a model organism for studying aging processes and evaluating the efficacy of various interventions. The compiled data provides a robust foundation for future research aimed at identifying effective strategies for lifespan extension and healthspan preservation. At the same time, the findings highlight the need for improved methodological consistency and the inclusion of multiple parameters within individual studies to achieve a more comprehensive understanding of oxidative stress in aging.

Taken together, the available evidence supports the use of *D. melanogaster* as a powerful model organism for aging research, particularly in evaluating the effects of dietary, genetic, and pharmacological interventions. The data also emphasize the importance of adopting standardized methodologies and incorporating multiple physiological and molecular markers within studies to achieve more comprehensive and reproducible results.

## Future Directions

Future studies should aim to overcome the current methodological and conceptual limitations by adopting a more integrative and standardized research framework. Harmonized experimental protocols that simultaneously assess both direct enzymatic ROS biomarkers (such as SOD, CAT, and GPx) and indirect oxidative damage markers (such as MDA) would improve comparability between studies and allow for a more reliable synthesis of results.

A broader perspective on aging in *Drosophila* also requires considering the complex interactions between oxidative stress, metabolism, and cellular signaling. Rather than focusing exclusively on individual antioxidant enzymes, future research should explore how coordinated regulatory networks influence redox balance and longevity. Incorporating genetic, biochemical, and physiological approaches within the same experimental design would provide a more complete understanding of how oxidative stress contributes to functional decline with age.

It will also be important to include time-course analyses that follow redox and physiological parameters throughout the lifespan, clarifying whether specific interventions act to delay the onset of damage or to enhance stress resistance at particular life stages. Studies should additionally consider biological context, including sex, genotype, and dietary background, as these factors can substantially alter the response to antioxidant or dietary interventions.

Growing evidence points to the role of gut microbiota and systemic metabolism in shaping stress resilience and lifespan in *Drosophila*. Future research should therefore address how dietary, probiotic, or environmental factors influence the interplay between redox balance and host physiology. Integrative, multilevel approaches that connect biochemical, microbiological, and behavioral data are likely to provide new insights into the mechanisms linking oxidative stress to aging.

Finally, future research should place greater emphasis on cross-disciplinary and translational perspectives. Comparative analyses across species will help determine which aspects of oxidative stress regulation are conserved and most relevant to human health. By combining standardized experimental approaches, multiparameter analyses, and systems-level perspectives, future studies can strengthen the predictive and translational value of *Drosophila* as a model for investigating aging and redox homeostasis.

## Data Availability

Not applicable.
